# Bis(*N*,*N*-dimethyl­formamide-κ*O*)bis­(1-methyl­imidazole-2-carbaldehyde oximato-κ^2^ *N*,*O*)manganese(III) perchlorate

**DOI:** 10.1107/S1600536808034016

**Published:** 2008-10-25

**Authors:** Feng Wang, Jianming Zhang, Qirong Wang, Shiying Huang

**Affiliations:** a College of Chemistry, Central China Normal University, Wuhan, Hubei 430079, People’s Republic of China; b School of Chemistry and Materials Science, Xiaogan University, Xiaogan, Hubei 432000, People’s Republic of China; c Hubei Polytechnic Institute, Xiaogan, Hubei 432000, People’s Republic of China

## Abstract

In the title compound, [Mn(C_5_H_6_N_3_O)_2_(C_3_H_7_NO)]ClO_4_, the Mn^III^ atom lies on the inversion centre of the centrosymmetric complex cation and has a distorted octa­hedral coordination geometry, formed by two N atoms and two O atoms from two 1-methyl­imidazole-2-carbaldehyde oximate ligands and two O atoms from two dimethyl­formamide ligands. Perchlorate acts as a counterion to balance the charge. The crystal structure of the title compound is stabilized by C—H⋯O hydrogen-bonding inter­actions.

## Related literature

For related literature, see: Miyasaka *et al.* (2005 ▶); Saitoh *et al.* (2007 ▶).
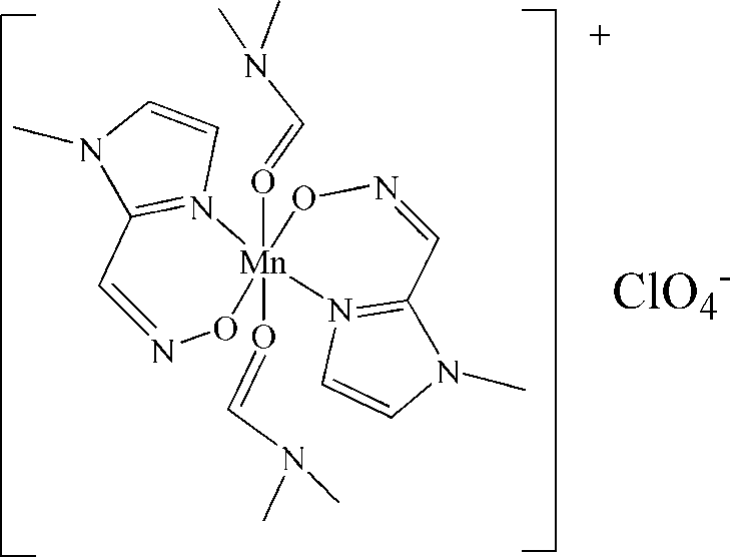


## Experimental

### 

#### Crystal data

[Mn(C_5_H_6_N_3_O)_2_(C_3_H_7_NO)]ClO_4_
*M* *_r_* = 548.84
Triclinic, 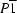

*a* = 7.6158 (11) Å
*b* = 12.324 (2) Å
*c* = 12.8600 (16) Å
α = 82.841 (10)°
β = 85.273 (11)°
γ = 80.471 (16)°
*V* = 1178.7 (3) Å^3^
*Z* = 2
Mo *K*α radiation
μ = 0.73 mm^−1^
*T* = 298 (2) K
0.23 × 0.20 × 0.10 mm

#### Data collection

Bruker SMART CCD area-detector diffractometer
Absorption correction: multi-scan (*SADABS*; Bruker, 2001 ▶) *T*_min_ = 0.850, *T*_max_ = 0.930
9397 measured reflections
4101 independent reflections
3191 reflections with *I* > 2σ(*I*)
*R*_int_ = 0.039

#### Refinement

*R*[*F* ^2^ > 2σ(*F* ^2^)] = 0.056
*wR*(*F* ^2^) = 0.158
*S* = 1.08
4101 reflections
313 parameters
H-atom parameters constrained
Δρ_max_ = 0.40 e Å^−3^
Δρ_min_ = −0.29 e Å^−3^

### 

Data collection: *SMART* (Bruker, 2001 ▶); cell refinement: *SAINT* (Bruker, 2001 ▶); data reduction: *SAINT*; program(s) used to solve structure: *SHELXS97* (Sheldrick, 2008 ▶); program(s) used to refine structure: *SHELXL97* (Sheldrick, 2008 ▶); molecular graphics: *SHELXTL* (Sheldrick, 2008 ▶); software used to prepare material for publication: *SHELXTL*.

## Supplementary Material

Crystal structure: contains datablocks I, global. DOI: 10.1107/S1600536808034016/at2654sup1.cif

Structure factors: contains datablocks I. DOI: 10.1107/S1600536808034016/at2654Isup2.hkl

Additional supplementary materials:  crystallographic information; 3D view; checkCIF report

## Figures and Tables

**Table 1:** Selected geometric parameters (Å, °)

Mn1—O1	1.886 (3)
Mn1—O2	1.893 (3)
Mn1—N4	1.997 (3)
Mn1—N2	2.002 (3)
Mn1—O4	2.204 (3)
Mn1—O3	2.443 (3)

**Table 2:** Hydrogen-bond geometry (Å, °)

*D*—H⋯*A*	*D*—H	H⋯*A*	*D*⋯*A*	*D*—H⋯*A*
C2—H2⋯O7^i^	0.93	2.54	3.433 (6)	161
C5—H5⋯O7^ii^	0.93	2.60	3.402 (6)	145
C12—H12*A*⋯O8^ii^	0.96	2.49	3.399 (6)	157
C13—H13*C*⋯O5^iii^	0.96	2.40	3.261 (7)	150
C14—H14⋯O6	0.93	2.59	3.477 (6)	161

Symmetry codes: (i) 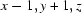; (ii) 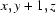; (iii) 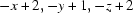.

**Fig. 1.**
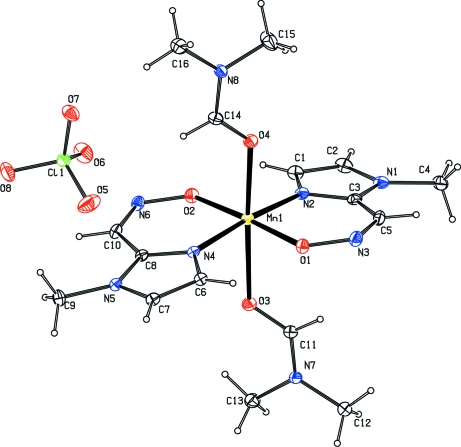
The molecular structure of (I), showing ellipsoids at the 50% probability level.

**Fig. 2.**
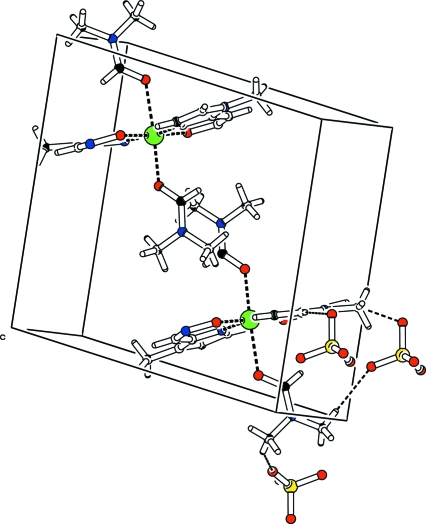
The hydrogen-bonding structure of the molecular packing diagram of (I).

## supplementary crystallographic information

### Comment

The metal complexes with the 2-((hydroxyimino)methyl)-1-methylimidazole and 2-((hydroxyimino)methyl)-1-ethylimidazole had some good activities (Miyasaka *et al.*, 2005; Saitoh *et al.*, 2007). Herein, we report the crystal structure of such a novel compound, [Mn(C_5_H_6_N_3_O)_2_(C_3_H_7_NO)](ClO_4_), (I).

The molecular structure of (I) is shown in Fig.1. The Mn atom lying on the inversion center of the centrosymmetric cation has a distorted octahedral geometry and is coordinated by two N atoms and two O atoms (O3,O4) from two 2-((hydroxyimino)methyl)-1-methylimidazole ligands, which lie in the equatorial plane, with the torsional angle O1—N2—O2—N4 = -2.19°, and two O atoms from two *N*',*N*-dimthylformamide molecules occupy the axial sites, which is nearly linear [O3—Mn—O4 = 176.62 (10)°] (Table 1). The distance from Mn to the equatorial plane is 0.0828 (15) Å. O3 and O4 are far away from the equatorial plane, with the mean distance 2.32 Å. As shown in Fig. 2, an organic cation layer is linked to an inorganic anionic layer through a series of C—H···O hydrogen bonding interactions (Table 2). In the structure, there are not π-π interactions.

### Experimental

[Mn(C_5_H_6_N_3_O)_2 _(C_3_H_7_NO)](ClO_4_) was prepared as followings: to a solution of 2-((Hydroxyimino)methyl)-1-methylimidazole 0.50 g(4 mmol) in DMF(25 mL) and triethylamine (0.05 mL) was added Mn(ClO_4_)_2 _(0.724 g, 2.0 mmol). After the mixture was stirred for a one hour, the solution was filtered. The filtrate was kept for several days at ambient temperature, and brown block crystals were obtained.

### Refinement

H atoms on C atoms were placed in geometrically idealized positions and refined in riding model, with C–H = 0.93 or 0.96 Å and *U*_iso_(H) = 1.2 or 1.5U_eq_(C).

### Crystal data

**Table:**

[Mn(C_5_H_6_N_3_O)_2_(C_3_H_7_NO)]ClO_4_	*Z* = 2
*M**_r_* = 548.84	*F*(000) = 568
Triclinic, *P*1	*D*_x_ = 1.546 Mg m^−^^3^
Hall symbol: -P 1	Mo *K*α radiation, λ = 0.71073 Å
*a* = 7.6158 (11) Å	Cell parameters from 2239 reflections
*b* = 12.324 (2) Å	θ = 2.5–22.4°
*c* = 12.8600 (16) Å	µ = 0.73 mm^−^^1^
α = 82.841 (10)°	*T* = 298 K
β = 85.273 (11)°	Block, brown
γ = 80.471 (16)°	0.23 × 0.20 × 0.10 mm
*V* = 1178.7 (3) Å^3^	

### Data collection

**Table:**

Bruker SMART CCD area-detector diffractometer	4101 independent reflections
Radiation source: fine-focus sealed tube	3191 reflections with *I* > 2σ(*I*)
graphite	*R*_int_ = 0.039
φ and ω scans	θ_max_ = 25.0°, θ_min_ = 1.6°
Absorption correction: multi-scan (SADABS; Bruker, 2001)	*h* = −8→9
*T*_min_ = 0.850, *T*_max_ = 0.930	*k* = −14→12
9397 measured reflections	*l* = −15→15

### Refinement

**Table:**

Refinement on *F*^2^	Primary atom site location: structure-invariant direct methods
Least-squares matrix: full	Secondary atom site location: difference Fourier map
*R*[*F*^2^ > 2σ(*F*^2^)] = 0.056	Hydrogen site location: inferred from neighbouring sites
*wR*(*F*^2^) = 0.158	H-atom parameters constrained
*S* = 1.08	*w* = 1/[σ^2^(*F*_o_^2^) + (0.0845*P*)^2^ + 0.2454*P*] where *P* = (*F*_o_^2^ + 2*F*_c_^2^)/3
4101 reflections	(Δ/σ)_max_ = 0.002
313 parameters	Δρ_max_ = 0.40 e Å^−^^3^
0 restraints	Δρ_min_ = −0.29 e Å^−^^3^

### Special details

**Table:**

Geometry. All e.s.d.'s (except the e.s.d. in the dihedral angle between two l.s. planes) are estimated using the full covariance matrix. The cell e.s.d.'s are taken into account individually in the estimation of e.s.d.'s in distances, angles and torsion angles; correlations between e.s.d.'s in cell parameters are only used when they are defined by crystal symmetry. An approximate (isotropic) treatment of cell e.s.d.'s is used for estimating e.s.d.'s involving l.s. planes.
Refinement. Refinement of *F*^2^ against ALL reflections. The weighted *R*-factor *wR* and goodness of fit *S* are based on *F*^2^, conventional *R*-factors *R* are based on *F*, with *F* set to zero for negative *F*^2^. The threshold expression of *F*^2^ > σ(*F*^2^) is used only for calculating *R*-factors(gt) *etc*. and is not relevant to the choice of reflections for refinement. *R*-factors based on *F*^2^ are statistically about twice as large as those based on *F*, and *R*- factors based on ALL data will be even larger.

### Fractional atomic coordinates and isotropic or equivalent isotropic displacement parameters (Å^2^)

**Table:**

	*x*	*y*	*z*	*U*_iso_*/*U*_eq_	
Mn1	0.67156 (7)	0.67997 (4)	0.75744 (4)	0.0366 (2)	
C1	0.3414 (5)	0.8512 (4)	0.7017 (3)	0.0513 (11)	
H1	0.2579	0.8049	0.7250	0.062*	
C2	0.3042 (6)	0.9582 (4)	0.6627 (3)	0.0579 (12)	
H2	0.1912	0.9989	0.6537	0.070*	
C3	0.5955 (6)	0.9116 (3)	0.6626 (3)	0.0446 (10)	
C4	0.4845 (7)	1.1121 (3)	0.5958 (4)	0.0667 (14)	
H4A	0.5257	1.1481	0.6491	0.100*	
H4B	0.3719	1.1521	0.5742	0.100*	
H4C	0.5699	1.1104	0.5366	0.100*	
C5	0.7840 (6)	0.9159 (4)	0.6449 (3)	0.0529 (11)	
H5	0.8146	0.9804	0.6076	0.063*	
C6	0.9974 (5)	0.5219 (3)	0.8403 (3)	0.0389 (9)	
H6	1.0818	0.5646	0.8096	0.047*	
C7	1.0311 (5)	0.4261 (3)	0.9042 (3)	0.0415 (9)	
H7	1.1425	0.3912	0.9251	0.050*	
C8	0.7428 (5)	0.4639 (3)	0.8846 (3)	0.0358 (9)	
C9	0.8461 (6)	0.2892 (3)	1.0015 (3)	0.0533 (11)	
H9A	0.7266	0.2973	1.0330	0.080*	
H9B	0.9294	0.2769	1.0555	0.080*	
H9C	0.8660	0.2272	0.9612	0.080*	
C10	0.5576 (5)	0.4523 (3)	0.8923 (3)	0.0462 (10)	
H10	0.5274	0.3893	0.9328	0.055*	
C11	0.6945 (5)	0.8367 (3)	0.9405 (3)	0.0431 (10)	
H11	0.7584	0.8692	0.8836	0.052*	
C12	0.7615 (6)	0.9785 (4)	1.0387 (3)	0.0573 (12)	
H12A	0.8234	1.0007	0.9734	0.086*	
H12B	0.8445	0.9599	1.0928	0.086*	
H12C	0.6711	1.0384	1.0570	0.086*	
C13	0.5781 (6)	0.8394 (4)	1.1206 (3)	0.0624 (13)	
H13A	0.5095	0.7868	1.1020	0.094*	
H13B	0.4993	0.8994	1.1488	0.094*	
H13C	0.6591	0.8038	1.1724	0.094*	
C14	0.7324 (5)	0.5286 (4)	0.5742 (3)	0.0497 (11)	
H14	0.7438	0.4704	0.6278	0.060*	
C15	0.7250 (11)	0.5901 (4)	0.3915 (4)	0.109 (3)	
H15A	0.6469	0.6535	0.4141	0.164*	
H15B	0.6748	0.5641	0.3350	0.164*	
H15C	0.8393	0.6104	0.3680	0.164*	
C16	0.7802 (8)	0.3905 (4)	0.4514 (4)	0.0720 (15)	
H16A	0.7721	0.3403	0.5143	0.108*	
H16B	0.8976	0.3759	0.4177	0.108*	
H16C	0.6935	0.3805	0.4047	0.108*	
N1	0.4629 (5)	0.9964 (3)	0.6388 (3)	0.0507 (9)	
N2	0.5233 (4)	0.8215 (3)	0.7014 (2)	0.0416 (8)	
N3	0.9150 (5)	0.8411 (3)	0.6746 (3)	0.0547 (9)	
N4	0.8164 (4)	0.5454 (2)	0.8285 (2)	0.0355 (7)	
N5	0.8711 (4)	0.3896 (3)	0.9328 (2)	0.0382 (7)	
N6	0.4281 (4)	0.5195 (3)	0.8494 (3)	0.0487 (8)	
N7	0.6785 (4)	0.8821 (3)	1.0277 (2)	0.0429 (8)	
N8	0.7459 (5)	0.5038 (3)	0.4778 (2)	0.0471 (8)	
O1	0.8785 (3)	0.7471 (2)	0.7320 (2)	0.0478 (7)	
O2	0.4629 (3)	0.6147 (2)	0.7914 (2)	0.0508 (7)	
O3	0.6303 (4)	0.7528 (2)	0.9275 (2)	0.0523 (7)	
O4	0.7056 (4)	0.6244 (2)	0.5996 (2)	0.0528 (7)	
Cl1	0.90018 (14)	0.19042 (8)	0.72382 (8)	0.0490 (3)	
O5	1.0504 (6)	0.2250 (4)	0.7606 (4)	0.1166 (16)	
O6	0.7487 (5)	0.2738 (3)	0.7265 (3)	0.0903 (12)	
O7	0.9418 (5)	0.1610 (3)	0.6206 (2)	0.0731 (10)	
O8	0.8620 (5)	0.0947 (3)	0.7917 (3)	0.0839 (11)	

### Atomic displacement parameters (Å^2^)

**Table:**

	*U*^11^	*U*^22^	*U*^33^	*U*^12^	*U*^13^	*U*^23^
Mn1	0.0330 (3)	0.0354 (4)	0.0413 (3)	−0.0012 (2)	−0.0041 (2)	−0.0077 (3)
C1	0.038 (2)	0.058 (3)	0.055 (2)	0.004 (2)	−0.0040 (18)	−0.010 (2)
C2	0.048 (3)	0.065 (3)	0.053 (2)	0.019 (2)	−0.007 (2)	−0.009 (2)
C3	0.053 (2)	0.041 (2)	0.042 (2)	0.002 (2)	−0.0120 (18)	−0.0156 (18)
C4	0.101 (4)	0.037 (3)	0.058 (3)	0.010 (2)	−0.026 (3)	−0.006 (2)
C5	0.060 (3)	0.038 (2)	0.063 (3)	−0.015 (2)	−0.010 (2)	0.000 (2)
C6	0.032 (2)	0.040 (2)	0.045 (2)	−0.0012 (17)	−0.0015 (16)	−0.0104 (18)
C7	0.030 (2)	0.044 (2)	0.050 (2)	−0.0010 (18)	−0.0050 (17)	−0.0114 (19)
C8	0.033 (2)	0.032 (2)	0.0417 (19)	0.0022 (16)	−0.0043 (15)	−0.0125 (17)
C9	0.052 (3)	0.041 (2)	0.061 (3)	−0.002 (2)	0.000 (2)	0.006 (2)
C10	0.041 (2)	0.039 (2)	0.061 (2)	−0.0122 (19)	−0.0018 (19)	−0.008 (2)
C11	0.040 (2)	0.046 (3)	0.043 (2)	0.0006 (19)	−0.0045 (16)	−0.0091 (19)
C12	0.066 (3)	0.053 (3)	0.054 (2)	−0.015 (2)	−0.007 (2)	−0.006 (2)
C13	0.071 (3)	0.075 (3)	0.046 (2)	−0.026 (3)	0.008 (2)	−0.014 (2)
C14	0.051 (2)	0.055 (3)	0.042 (2)	−0.003 (2)	−0.0034 (18)	−0.008 (2)
C15	0.214 (8)	0.056 (4)	0.049 (3)	−0.001 (4)	0.007 (4)	−0.009 (3)
C16	0.108 (4)	0.045 (3)	0.059 (3)	0.011 (3)	−0.011 (3)	−0.016 (2)
N1	0.062 (2)	0.042 (2)	0.0467 (19)	0.0087 (18)	−0.0146 (16)	−0.0158 (16)
N2	0.0407 (19)	0.040 (2)	0.0439 (17)	0.0022 (15)	−0.0072 (14)	−0.0124 (15)
N3	0.050 (2)	0.051 (2)	0.065 (2)	−0.0155 (19)	−0.0046 (17)	−0.0029 (19)
N4	0.0321 (16)	0.0362 (18)	0.0394 (16)	−0.0043 (14)	−0.0039 (13)	−0.0098 (14)
N5	0.0349 (17)	0.0350 (18)	0.0441 (17)	−0.0014 (14)	−0.0020 (13)	−0.0080 (14)
N6	0.0378 (19)	0.047 (2)	0.063 (2)	−0.0114 (17)	−0.0043 (16)	−0.0048 (18)
N7	0.0455 (19)	0.047 (2)	0.0383 (17)	−0.0110 (16)	−0.0037 (14)	−0.0052 (15)
N8	0.063 (2)	0.039 (2)	0.0374 (17)	−0.0003 (17)	−0.0038 (15)	−0.0041 (15)
O1	0.0418 (16)	0.0430 (17)	0.0573 (16)	−0.0054 (13)	−0.0033 (12)	−0.0020 (14)
O2	0.0359 (15)	0.0536 (19)	0.0620 (17)	−0.0066 (13)	−0.0089 (13)	0.0001 (15)
O3	0.0614 (19)	0.0491 (18)	0.0478 (16)	−0.0076 (15)	−0.0047 (13)	−0.0112 (14)
O4	0.071 (2)	0.0438 (17)	0.0434 (15)	−0.0033 (15)	−0.0003 (13)	−0.0156 (13)
Cl1	0.0570 (7)	0.0428 (6)	0.0473 (5)	−0.0056 (5)	−0.0017 (4)	−0.0092 (5)
O5	0.098 (3)	0.150 (4)	0.124 (3)	−0.060 (3)	−0.011 (3)	−0.046 (3)
O6	0.101 (3)	0.067 (2)	0.088 (2)	0.027 (2)	0.002 (2)	−0.008 (2)
O7	0.102 (3)	0.062 (2)	0.0477 (17)	0.0068 (19)	0.0013 (17)	−0.0089 (15)
O8	0.113 (3)	0.067 (2)	0.064 (2)	−0.015 (2)	0.0083 (19)	0.0157 (18)

### Geometric parameters (Å, °)

**Table:**

Mn1—O1	1.886 (3)	C9—H9C	0.9600
Mn1—O2	1.893 (3)	C10—N6	1.292 (5)
Mn1—N4	1.997 (3)	C10—H10	0.9300
Mn1—N2	2.002 (3)	C11—O3	1.249 (5)
Mn1—O4	2.204 (3)	C11—N7	1.304 (5)
Mn1—O3	2.443 (3)	C11—H11	0.9300
C1—C2	1.344 (6)	C12—N7	1.461 (5)
C1—N2	1.372 (5)	C12—H12A	0.9600
C1—H1	0.9300	C12—H12B	0.9600
C2—N1	1.367 (6)	C12—H12C	0.9600
C2—H2	0.9300	C13—N7	1.455 (5)
C3—N2	1.343 (5)	C13—H13A	0.9600
C3—N1	1.352 (5)	C13—H13B	0.9600
C3—C5	1.443 (6)	C13—H13C	0.9600
C4—N1	1.493 (5)	C14—O4	1.244 (5)
C4—H4A	0.9600	C14—N8	1.306 (5)
C4—H4B	0.9600	C14—H14	0.9300
C4—H4C	0.9600	C15—N8	1.437 (6)
C5—N3	1.289 (5)	C15—H15A	0.9600
C5—H5	0.9300	C15—H15B	0.9600
C6—C7	1.351 (5)	C15—H15C	0.9600
C6—N4	1.377 (5)	C16—N8	1.455 (5)
C6—H6	0.9300	C16—H16A	0.9600
C7—N5	1.375 (5)	C16—H16B	0.9600
C7—H7	0.9300	C16—H16C	0.9600
C8—N4	1.335 (5)	N3—O1	1.350 (4)
C8—N5	1.356 (4)	N6—O2	1.362 (4)
C8—C10	1.436 (5)	Cl1—O6	1.413 (3)
C9—N5	1.458 (5)	Cl1—O7	1.416 (3)
C9—H9A	0.9600	Cl1—O5	1.420 (4)
C9—H9B	0.9600	Cl1—O8	1.433 (3)
			
O1—Mn1—O2	176.56 (11)	H12A—C12—H12B	109.5
O1—Mn1—N4	89.45 (12)	N7—C12—H12C	109.5
O2—Mn1—N4	89.88 (12)	H12A—C12—H12C	109.5
O1—Mn1—N2	90.13 (13)	H12B—C12—H12C	109.5
O2—Mn1—N2	90.18 (13)	N7—C13—H13A	109.5
N4—Mn1—N2	173.75 (11)	N7—C13—H13B	109.5
O1—Mn1—O4	91.23 (11)	H13A—C13—H13B	109.5
O2—Mn1—O4	92.20 (12)	N7—C13—H13C	109.5
N4—Mn1—O4	95.89 (11)	H13A—C13—H13C	109.5
N2—Mn1—O4	90.35 (11)	H13B—C13—H13C	109.5
O1—Mn1—O3	87.46 (11)	O4—C14—N8	124.9 (4)
O2—Mn1—O3	89.13 (11)	O4—C14—H14	117.6
N4—Mn1—O3	87.21 (10)	N8—C14—H14	117.6
N2—Mn1—O3	86.54 (10)	N8—C15—H15A	109.5
O4—Mn1—O3	176.62 (10)	N8—C15—H15B	109.5
C2—C1—N2	108.6 (4)	H15A—C15—H15B	109.5
C2—C1—H1	125.7	N8—C15—H15C	109.5
N2—C1—H1	125.7	H15A—C15—H15C	109.5
C1—C2—N1	107.5 (4)	H15B—C15—H15C	109.5
C1—C2—H2	126.3	N8—C16—H16A	109.5
N1—C2—H2	126.3	N8—C16—H16B	109.5
N2—C3—N1	108.9 (4)	H16A—C16—H16B	109.5
N2—C3—C5	125.5 (4)	N8—C16—H16C	109.5
N1—C3—C5	125.5 (4)	H16A—C16—H16C	109.5
N1—C4—H4A	109.5	H16B—C16—H16C	109.5
N1—C4—H4B	109.5	C3—N1—C2	107.8 (4)
H4A—C4—H4B	109.5	C3—N1—C4	126.4 (4)
N1—C4—H4C	109.5	C2—N1—C4	125.7 (4)
H4A—C4—H4C	109.5	C3—N2—C1	107.2 (4)
H4B—C4—H4C	109.5	C3—N2—Mn1	122.2 (3)
N3—C5—C3	127.9 (4)	C1—N2—Mn1	130.4 (3)
N3—C5—H5	116.1	C5—N3—O1	118.6 (3)
C3—C5—H5	116.1	C8—N4—C6	107.2 (3)
C7—C6—N4	108.3 (3)	C8—N4—Mn1	122.6 (2)
C7—C6—H6	125.9	C6—N4—Mn1	129.8 (3)
N4—C6—H6	125.9	C8—N5—C7	107.1 (3)
C6—C7—N5	107.7 (3)	C8—N5—C9	127.0 (3)
C6—C7—H7	126.2	C7—N5—C9	125.9 (3)
N5—C7—H7	126.2	C10—N6—O2	119.1 (3)
N4—C8—N5	109.7 (3)	C11—N7—C13	122.0 (4)
N4—C8—C10	126.5 (3)	C11—N7—C12	122.2 (3)
N5—C8—C10	123.7 (4)	C13—N7—C12	115.8 (3)
N5—C9—H9A	109.5	C14—N8—C15	120.2 (4)
N5—C9—H9B	109.5	C14—N8—C16	123.1 (4)
H9A—C9—H9B	109.5	C15—N8—C16	116.7 (3)
N5—C9—H9C	109.5	N3—O1—Mn1	133.5 (2)
H9A—C9—H9C	109.5	N6—O2—Mn1	134.0 (2)
H9B—C9—H9C	109.5	C11—O3—Mn1	120.4 (2)
N6—C10—C8	127.3 (4)	C14—O4—Mn1	129.3 (3)
N6—C10—H10	116.3	O6—Cl1—O7	110.6 (2)
C8—C10—H10	116.3	O6—Cl1—O5	111.4 (3)
O3—C11—N7	124.9 (4)	O7—Cl1—O5	109.5 (2)
O3—C11—H11	117.6	O6—Cl1—O8	108.6 (2)
N7—C11—H11	117.6	O7—Cl1—O8	108.9 (2)
N7—C12—H12A	109.5	O5—Cl1—O8	107.7 (3)
N7—C12—H12B	109.5		
			
N2—C1—C2—N1	−0.6 (4)	O3—Mn1—N4—C8	81.1 (3)
N2—C3—C5—N3	−9.7 (7)	O1—Mn1—N4—C6	−3.9 (3)
N1—C3—C5—N3	173.1 (4)	O2—Mn1—N4—C6	179.5 (3)
N4—C6—C7—N5	−0.2 (4)	O4—Mn1—N4—C6	87.3 (3)
N4—C8—C10—N6	−2.3 (6)	O3—Mn1—N4—C6	−91.4 (3)
N5—C8—C10—N6	179.7 (4)	N4—C8—N5—C7	−0.6 (4)
N2—C3—N1—C2	−0.3 (4)	C10—C8—N5—C7	177.7 (3)
C5—C3—N1—C2	177.2 (4)	N4—C8—N5—C9	179.7 (3)
N2—C3—N1—C4	178.6 (3)	C10—C8—N5—C9	−1.9 (5)
C5—C3—N1—C4	−3.8 (6)	C6—C7—N5—C8	0.5 (4)
C1—C2—N1—C3	0.6 (4)	C6—C7—N5—C9	−179.9 (3)
C1—C2—N1—C4	−178.4 (3)	C8—C10—N6—O2	−1.7 (6)
N1—C3—N2—C1	0.0 (4)	O3—C11—N7—C13	−1.3 (6)
C5—C3—N2—C1	−177.6 (3)	O3—C11—N7—C12	178.2 (4)
N1—C3—N2—Mn1	−175.1 (2)	O4—C14—N8—C15	−1.6 (7)
C5—C3—N2—Mn1	7.4 (5)	O4—C14—N8—C16	179.0 (4)
C2—C1—N2—C3	0.4 (4)	C5—N3—O1—Mn1	16.5 (5)
C2—C1—N2—Mn1	174.9 (3)	N4—Mn1—O1—N3	171.2 (3)
O1—Mn1—N2—C3	2.4 (3)	N2—Mn1—O1—N3	−15.0 (3)
O2—Mn1—N2—C3	178.9 (3)	O3—Mn1—O1—N3	−101.5 (3)
O4—Mn1—N2—C3	−88.9 (3)	C10—N6—O2—Mn1	−1.6 (5)
O3—Mn1—N2—C3	89.8 (3)	N4—Mn1—O2—N6	5.5 (3)
O1—Mn1—N2—C1	−171.4 (3)	N2—Mn1—O2—N6	−168.3 (3)
O2—Mn1—N2—C1	5.1 (3)	O4—Mn1—O2—N6	101.4 (3)
O4—Mn1—N2—C1	97.3 (3)	O3—Mn1—O2—N6	−81.7 (3)
O3—Mn1—N2—C1	−84.0 (3)	N7—C11—O3—Mn1	179.5 (3)
C3—C5—N3—O1	−2.1 (6)	O1—Mn1—O3—C11	25.0 (3)
N5—C8—N4—C6	0.5 (4)	O2—Mn1—O3—C11	−155.5 (3)
C10—C8—N4—C6	−177.8 (3)	N4—Mn1—O3—C11	114.6 (3)
N5—C8—N4—Mn1	−173.4 (2)	N2—Mn1—O3—C11	−65.2 (3)
C10—C8—N4—Mn1	8.3 (5)	N8—C14—O4—Mn1	177.9 (3)
C7—C6—N4—C8	−0.2 (4)	O1—Mn1—O4—C14	117.2 (4)
C7—C6—N4—Mn1	173.1 (2)	O2—Mn1—O4—C14	−62.5 (4)
O1—Mn1—N4—C8	168.6 (3)	N4—Mn1—O4—C14	27.6 (4)
O2—Mn1—N4—C8	−8.1 (3)	N2—Mn1—O4—C14	−152.7 (4)
O4—Mn1—N4—C8	−100.3 (3)		

### Hydrogen-bond geometry (Å, °)

**Table:**

*D*—H···*A*	*D*—H	H···*A*	*D*···*A*	*D*—H···*A*
C2—H2···O7^i^	0.93	2.54	3.433 (6)	161
C5—H5···O7^ii^	0.93	2.60	3.402 (6)	145
C12—H12A···O8^ii^	0.96	2.49	3.399 (6)	157
C13—H13A···O3	0.96	2.41	2.797 (5)	103
C13—H13C···O5^iii^	0.96	2.40	3.261 (7)	150
C14—H14···O6	0.93	2.59	3.477 (6)	161

Symmetry codes: (i) *x*−1, *y*+1, *z*; (ii) *x*, *y*+1, *z*; (iii) −*x*+2, −*y*+1, −*z*+2.
